# Urinary tract infections in diabetes: clinical significance and management approaches

**DOI:** 10.3389/abp.2026.17198

**Published:** 2026-08-17

**Authors:** Beata Zalewska-Piątek, Michalina Nagórka, Rafał Piątek

**Affiliations:** Department of Biotechnology and Microbiology, Chemical Faculty, Gdańsk University of Technology, Gdańsk, Poland

**Keywords:** diabetes mellitus, glucosuria, glycemic control, host–pathogen interactions, hyperglycemia

## Abstract

Diabetes mellitus is a significant risk factor for urinary tract infections (UTIs), which occur more frequently, have a more severe course, and show a greater tendency to recur in affected individuals. Metabolic disturbances, particularly hyperglycemia and glucosuria, promote bacterial growth while simultaneously impairing innate and adaptive immune responses. In addition, diabetes-related complications, such as autonomic neuropathy, contribute to bladder dysfunction and urinary stasis, further facilitating bacterial colonization. Epidemiological evidence indicates a two- to four-fold increased risk of UTIs in patients with diabetes, along with a high prevalence of asymptomatic bacteriuria and recurrent infections. A key feature of this relationship is its bidirectional nature. UTIs can exacerbate glycemic dysregulation by triggering stress responses and increasing insulin resistance, leading to transient or sustained hyperglycemia. Uropathogenic *Escherichia coli* plays a critical role in the pathogenesis of UTIs, with hyperglycemic conditions further enhancing bacterial adhesion, biofilm formation, and immune evasion. Effective management requires simultaneous control of infection and optimization of glycemic control, particularly in the context of rising antimicrobial resistance. Emerging therapeutic strategies include targeting bacterial adhesion, disrupting biofilms, and modulating host immune responses. Improved understanding of host–pathogen interactions may enable the development of more effective preventive and therapeutic approaches in this high-risk population.

## Introduction

Diabetes mellitus correlates with a broad range of complications, among which urinary tract infections (UTIs) are particularly common and clinically significant. Impaired glucose regulation and alterations in host defense mechanisms render individuals with diabetes more susceptible to infections, creating a complex interplay between metabolic and infectious processes. Hyperglycemia refers to elevated blood glucose levels above physiological ranges, while hypoglycemia is defined as abnormally low blood glucose levels, often accompanied by symptoms such as sweating, confusion, and palpitations (American Diabetes Association Professional Practice Committee et al., 2024). Both conditions represent extremes of dysglycemia commonly observed in diabetes mellitus. Glucosuria describes urinary glucose excretion that typically occurs once blood glucose levels exceed the renal reabsorption threshold, although it may also arise in conditions associated with impaired tubular reabsorption (Geerlings et al., 2014). These metabolic disturbances may contribute to an environment that predisposes diabetic individuals to infections.

In diabetic individuals, UTIs represent a significant clinical problem due to their increased frequency, higher recurrence rates, and often more complicated course. Several biological mechanisms contribute to this heightened susceptibility. Chronic hyperglycemia is associated with glucosuria, creating a nutrient-rich urinary environment for bacterial growth, while simultaneously impairing leukocyte function and cytokine-mediated immune responses (Kamei and Yamamoto, 2021). In addition, diabetic autonomic neuropathy may lead to incomplete bladder emptying and urinary stasis, further facilitating bacterial colonization (Ahmad et al., 2020). Alterations in innate immune responses and microenvironmental changes in the urinary tract also promote bacterial adhesion to epithelial surfaces and biofilm formation (Geerlings, 2008; Nitzan et al., 2015).

Overall, metabolic and immunological disturbances in diabetes significantly increase the risk of UTIs and their complications. Therefore, a better understanding of this relationship is crucial for optimizing patient care and reducing infection-related morbidity in diabetic populations.

## Epidemiological and biological perspectives of diabetes as a risk factor for UTIs

Epidemiological evidence consistently demonstrates a higher risk of UTIs among patients with diabetes. Diabetes has been associated with a two- to four-fold increase in both the incidence and recurrence of UTIs. This increased vulnerability is largely attributed to glucosuria-driven bacterial growth, impaired innate immune defenses, and altered epithelial barrier function (Geerlings, 2008; Nitzan et al., 2015). Population-based data further highlight this relationship. For example, a meta-analysis including 15 studies and 827,948 individuals with type 2 diabetes reported an estimated UTI prevalence of approximately 11.5% (Salari et al., 2022). Additionally, asymptomatic bacteriuria (ASB) appears to be relatively common in this population, with a reported prevalence of 23.7% among patients with type 2 diabetes (Dai et al., 2023). However, more recent studies suggest that the burden may be even higher in certain populations, with prevalence reaching up to 39.3% and an overall risk nearly doubled compared to non-diabetic individuals (Ahmed et al., 2023; Abdelaziz et al., 2026).

Beyond single episodes, patients with diabetes are at increased risk of recurrent UTIs, with reported recurrence rates ranging from approximately 23%–37% (Papp and Zimmern, 2023). Diabetes has been identified as an independent risk factor for recurrence, and affected individuals are more likely to progress from an initial episode of cystitis to recurrent disease (Ackerman et al., 2026). Contributing factors may include bladder dysfunction, urinary incontinence, obesity, and long-standing disease, although their relative impact varies across studies (Papp and Zimmern, 2023).

Recent epidemiological studies have provided a more comprehensive characterization of UTIs in patients with diabetes. Compared with non-diabetic individuals, patients with diabetes exhibit a significantly higher prevalence of culture-confirmed UTIs, with reported rates ranging from approximately 40%–45% versus 28%–30% in non-diabetic controls (Abdelaziz et al., 2026). Individuals over 45 years of age appear to be particularly susceptible to infection. Furthermore, although both type 1 and type 2 diabetes increase the risk of UTIs, available evidence suggests that patients with type 2 diabetes experience UTIs more frequently than those with type 1 diabetes, most likely because of the higher prevalence of obesity, insulin resistance, poorer glycemic control, and chronic diabetic complications rather than diabetes type itself (Geerlings, 2008; Nitzan et al., 2015; Abdelaziz et al., 2026).

Although uropathogenic *Escherichia coli* (UPEC) remains the predominant etiological agent of uncomplicated UTIs in patients with and without diabetes, the microbiological spectrum broadens considerably in more complex infections. Across different studies, *E. coli* accounted for approximately 60%–71% of isolates, whereas *Klebsiella pneumoniae*, *Proteus mirabilis*, *Pseudomonas aeruginosa*, *Enterococcus spp*., *Enterobacter spp*., and, less frequently, *Candida albicans* were also frequently identified in patients with diabetes (Ahmad et al., 2020; Pishdad et al., 2024; Abdelaziz et al., 2026). Moreover, patients with diabetes exhibit a higher prevalence of infections caused by antimicrobial-resistant pathogens, including extended-spectrum β-lactamase (ESBL)-producing *E. coli* and *K. pneumoniae*, further complicating the management of UTIs (Ahmad et al., 2020; Papp and Zimmern, 2023).

The distribution of uropathogens depends not only on the presence of diabetes but also on the type and complexity of infection. While *E. coli* predominates in uncomplicated cystitis, *non-E. coli* pathogens are isolated more frequently in recurrent, complicated, healthcare-associated, and catheter-associated UTIs. In particular, *K. pneumoniae* and *P. mirabilis* are commonly associated with complicated infections, whereas *P. aeruginosa* and *Enterococcus spp*. are more frequently recovered from healthcare-associated or catheter-associated UTIs. *Candida spp*. should also be considered an important opportunistic pathogen in patients with poorly controlled diabetes, prolonged antibiotic exposure, urinary catheterization, or other predisposing conditions (Kamei and Yamamoto, 2021; Pishdad et al., 2024).

The broader spectrum of uropathogens observed in patients with diabetes is accompanied by a higher incidence of recurrent and complicated UTIs, including pyelonephritis, emphysematous cystitis, emphysematous pyelonephritis, renal abscesses, and urosepsis (Kamei and Yamamoto, 2021). These clinical manifestations are primarily attributed to hyperglycemia-induced impairment of innate immune responses, glucosuria, autonomic neuropathy leading to urinary stasis, and chronic inflammation rather than diabetes type itself (Pishdad et al., 2024).

Although epidemiological studies consistently demonstrate an increased incidence and recurrence of UTIs in patients with diabetes, interpretation of these findings remains challenging due to the observational nature of most available evidence and substantial heterogeneity in study populations, diagnostic criteria, and definitions of recurrent UTIs (Geerlings, 2008; Nitzan et al., 2015; Storme et al., 2019; Salari et al., 2022; Papp and Zimmern, 2023; Sorescu et al., 2024). Consequently, the relative contribution of hyperglycemia, disease duration, obesity, diabetic neuropathy, renal impairment, and other comorbidities remains difficult to determine (Nitzan et al., 2015; Papp and Zimmern, 2023).

From a clinical perspective, UTIs in patients with diabetes may present with typical symptoms such as dysuria, urinary frequency, and lower abdominal pain; however, atypical or less pronounced symptoms are also common, which may delay diagnosis (Storme et al., 2019; Kamei and Yamamoto, 2021). In recurrent cases, urinary urgency and abdominal discomfort may become more prominent. Importantly, UTIs associated with diabetes are more likely to progress to severe complications, including acute pyelonephritis, renal abscesses, emphysematous infections, and urosepsis (Kamei and Yamamoto, 2021; Ackerman et al., 2026). Together, these findings highlight the substantial burden of urinary infections in diabetic populations.

## Association between hyperglycemia and UTIs in patients with diabetes

Infections of the urinary tract are a frequent and clinically significant complication in patients with diabetes, as they can destabilize blood glucose levels and create a bidirectional interaction between infection and metabolic control. In individuals with diabetes mellitus, UTIs have an additional clinical significance because infections can exacerbate glycemic dysregulation (Nitzan et al., 2015; Lenherr et al., 2016). Diabetes itself constitutes an important host-related factor predisposing to UTIs, while infection can, in turn, worsen glycemic control, creating a bidirectional relationship between metabolic and infectious processes (Confederat et al., 2023; Abu-Humaidan et al., 2025; Wajid et al., 2025).

UTIs may contribute to transient or sustained hyperglycemia in individuals with diabetes through activation of the physiological stress response. During infection, the body releases counterregulatory hormones that increase hepatic glucose production and reduce insulin sensitivity, leading to elevated blood glucose levels (Dandona, 2004; Galindo et al., 2020; Tama et al., 2025; Wajid et al., 2025). In addition, inflammatory processes accompanying UTIs can aggravate insulin resistance, further worsening glycemic control in patients with diabetes (Geerlings and Hoepelman, 1999; Galindo et al., 2020). Persistent low-grade inflammation related to infection may intensify this effect, particularly in individuals with impaired renal function (Dandona, 2004; Galindo et al., 2020).

Clinically, unexplained increases in blood glucose should prompt consideration of a possible infection such as UTIs in patients with diabetes (Wajid et al., 2025). Management requires treatment of the underlying infection together with careful glycemic monitoring, as temporary adjustments in antidiabetic therapy and more frequent glucose measurements may be necessary during treatment (Galindo et al., 2020; March et al., 2023; American Diabetes Association Professional Practice Committee et al., 2024; Wajid et al., 2025). Patients with advanced kidney disease require particularly close monitoring because they may develop both hyperglycemia and hypoglycemia depending on the stage of renal impairment (Galindo et al., 2020).

Despite substantial experimental and clinical evidence supporting the association between hyperglycemia and increased susceptibility to UTIs, direct causal relationships remain difficult to establish. Most mechanistic evidence originates from experimental models, whereas human studies are predominantly observational (Geerlings and Hoepelman, 1999; Islam et al., 2022; Mohanty et al., 2022). Therefore, although hyperglycemia is widely considered an important contributing factor, its independent effect is often difficult to separate from coexisting diabetic complications, immune dysfunction, and urinary tract (Nitzan et al., 2015; Kamei and Yamamoto, 2021).

Overall, these interactions highlight the importance of recognizing infection as a potential trigger of glycemic instability and underscore the need for integrated management of both metabolic control and underlying infectious processes in patients with diabetes. To better understand this bidirectional relationship, it is important to consider why individuals with diabetes are more susceptible to UTIs in the first place.

## Clinical management and monitoring of UTI-associated hyperglycemia

Unexplained elevations in blood glucose levels in patients with diabetes should prompt consideration of an underlying infection, particularly UTIs, given their increased frequency, severity, and risk of complications in diabetes mellitus (Nitzan et al., 2015; Confederat et al., 2023). In managing UTI-associated hyperglycemia, prompt antimicrobial therapy tailored to culture results is recommended, especially since diabetic UTIs more often involve resistant pathogens and may require broader-spectrum antibiotics based on local susceptibility patterns (Confederat et al., 2023; Abu-Humaidan et al., 2025). Recent comparative evidence further indicates that antimicrobial resistance in diabetic UTIs is not only more prevalent but may also involve specific antibiotic classes. Abdelaziz et al. (2026) demonstrated significantly higher resistance among uropathogens isolated from diabetic patients to amoxicillin (85.0% vs. 67.5%), amoxicillin/clavulanic acid (57.5% vs. 37.5%), ciprofloxacin (38.8% vs. 20.0%), and ceftriaxone (25.0% vs. 7.5%) compared with isolates from non-diabetic individuals. Overall, resistance to at least one antimicrobial agent was detected in 92.5% of isolates from diabetic patients versus 75.0% of isolates from non-diabetic controls, with *E. coli* exhibiting the most pronounced difference between the groups. Nevertheless, these findings should not be interpreted as evidence that any specific antibiotic should be universally avoided in diabetic patients. Rather, they emphasize the importance of urine culture, antimicrobial susceptibility testing, and consideration of local resistance patterns when selecting empirical therapy, particularly in recurrent or complicated UTIs (Abdelaziz et al., 2026).

In the inpatient setting, frequent glucose monitoring and adherence to appropriate glycemic targets are essential (Pasquel et al., 2021). Current clinical practice guidelines recommend structured glucose monitoring and insulin-centered regimens to maintain glucose within target ranges and minimize complications such as infection progression or metabolic decompensation. Insulin remains the preferred modality for controlling significant hyperglycemia in hospitalized patients, with careful adjustment based on glucose trends and clinical status (Pasquel et al., 2021; Korytkowski et al., 2022). Additionally, poor glycemic control itself is linked to an increased frequency of UTIs, suggesting that optimizing blood glucose levels may reduce infection risk and improve outcomes (Lenherr et al., 2016). For patients with comorbid chronic kidney disease or other complications, individualized monitoring and adjustment of both antidiabetic therapy and antibiotic dosing are particularly important due to altered pharmacokinetics and fluctuating glycemic responses (Korytkowski et al., 2022).

Effective management of UTI-induced hyperglycemia requires a dual focus on rapid treatment of the infection and careful glycemic control (Pasquel et al., 2021; Korytkowski et al., 2022). Close monitoring, timely therapy adjustments, and attention to patient-specific factors are essential to prevent complications and improve outcomes.

## Mechanisms, clinical patterns, and modeling considerations of glucosuria in diabetes

Glucosuria reflects a disturbance in renal glucose handling closely tied to systemic glucose homeostasis. Under normal circumstances, the kidneys filter a large quantity of plasma glucose daily, yet virtually none appears in the final urine because nearly all filtered glucose is reabsorbed in the proximal tubule through sodium–glucose cotransporters, primarily SGLT2 with a smaller contribution by SGLT1 (Ghezzi et al., 2018; Sędzikowska and Szablewski, 2021). This coordinated transport prevents measurable glucosuria in healthy individuals, with urinary glucose concentrations typically below 0.5 mmol/L (≈0.09 g/L) and undetectable by standard clinical assays, thereby remaining insufficient to drive protein glycation (Liman and Jialal, 2026). By contrast, in patients with persistent hyperglycemia or those receiving SGLT2 inhibitor therapy, urinary glucose excretion can increase markedly. Hospitalized individuals with diabetes have been reported to excrete over 50 g/day of glucose, corresponding to urinary concentrations of approximately 5–25 mmol/L depending on urine output and hydration status (Monobe et al., 2021). This physiological mechanism has been therapeutically exploited through the development of SGLT2 inhibitors, which reduce glucose reabsorption in the proximal tubule and can increase urinary glucose excretion to over 60–70 g/day in patients with preserved renal function (Hu et al., 2022).

Beyond improving glycemic control, SGLT2 inhibitors confer substantial cardiovascular and renal benefits. However, their mechanism of action inducing glucosuria has raised concerns about a potentially increased risk of infections due to a more favorable environment for microbial growth (Carpenter, 2025; Dobashi et al., 2026). While evidence regarding UTIs remains inconsistent, randomized controlled trials and meta-analyses generally show no significant increase in risk. Observational studies, however, suggest only a modest elevation. The risk appears to be influenced more by factors such as impaired kidney function and prior genitourinary infections than by glycemic control alone (Yang et al., 2018; Zhang et al., 2025; Khan, 2026). These discrepancies may partly reflect differences in study design, patient populations, and underlying clinical characteristics. In contrast, there is consistent evidence of a markedly increased risk of genital infections more than threefold particularly in women and individuals with a history of such infections, although these are typically mild and respond well to standard treatment (Staplin et al., 2021; Pishdad et al., 2024; Wu et al., 2025). Importantly, the contribution of glucosuria itself to UTI susceptibility remains incompletely understood, as experimental studies supporting a role for urinary glucose in bacterial growth and UPEC virulence are not fully consistent with clinical observations from patients treated with SGLT2 inhibitors (Staplin et al., 2021; Islam et al., 2022; Pishdad et al., 2024).

In diabetes mellitus, chronic hyperglycemia can exceed the tubular reabsorptive capacity. Once plasma glucose concentrations surpass the tubular transport maximum historically termed the “renal threshold” glucose begins to spill into the urine, producing measurable glucosuria. The renal threshold varies between individuals and with renal function, but the basic mechanism remains that exceeding reabsorptive capacity results in glucosuria (Rave et al., 2006; Ghezzi et al., 2018; Tang et al., 2025). Notably, glucosuria demonstrates considerable interindividual variability, which is influenced by differences in glomerular filtration rate, the expression and function of renal glucose transporters, as well as genetic variation in the SLC5A2 gene encoding SGLT2. This contributes to the heterogeneity of urinary glucose levels observed in patients with diabetes (Zhou et al., 2023; Allaire et al., 2025; Tonin et al., 2025).

Taken together, these physiological and clinical observations support the concept that urinary glucose concentrations in individuals with significant hyperglycemia can span a wide spectrum. Although routine clinical testing tends to be semiquantitative, research studies demonstrate that diabetic glucosuria can reach tens of grams per day under sustained hyperglycemia or pharmacological SGLT2 inhibition (Rave et al., 2006; Hu et al., 2022). Within this context, models aiming to reproduce moderate glucosuria commonly employ glucose concentrations in the range of 0.1%–0.5% (1–5 g/L) to reflect urinary conditions observed in diabetes (Tonin et al., 2025).

This spectrum of glucosuria highlights the clinical relevance of monitoring urinary glucose in patients with diabetes, both for assessing glycemic control and for identifying risk factors for UTIs. Moreover, it provides a practical framework for experimental studies, enabling researchers to model clinically meaningful glucose exposure *in vitro*. Ultimately, integrating knowledge of glucosuria with patient-specific factors enhances both therapeutic decision-making and translational research in diabetes.

## UPEC pathogenesis in diabetes

UPEC are highly adaptive uropathogens whose virulence appears to be enhanced under hyperglycemic conditions, making patients with diabetes particularly susceptible to persistent and severe UTIs (Islam et al., 2022). In diabetes, metabolic and immunological alterations may facilitate bacterial colonization and modulate virulence, which may result in a complex interaction between bacterial pathogenicity and host metabolic dysregulation (Neupane et al., 2026).

The infection process begins with bacterial adhesion to uroepithelial cells mediated mainly by type 1 and P pili. The FimH adhesin, a tip subunit of type 1 pili, promotes bacterial internalization and the formation of intracellular bacterial communities, protecting bacteria from immune responses and antibiotics. P pili, particularly class II variants, are associated with upper urinary tract colonization and enhanced renal inflammation under diabetic conditions (Tseng et al., 2018). UPEC further express multiple virulence factors, including hemolysin A, cytotoxic necrotizing factor 1, the curli fiber-associated protein CsgA, serum resistance-associated protein TraT, and group 2 capsule-associated factors encoded by the genes *hlyA*, *cnf1*, *csgA*, *traT*, and *kpsMTII*, respectively, which contribute to tissue damage, biofilm formation, and immune evasion (Shahin et al., 2019).

**FIGURE 1:**
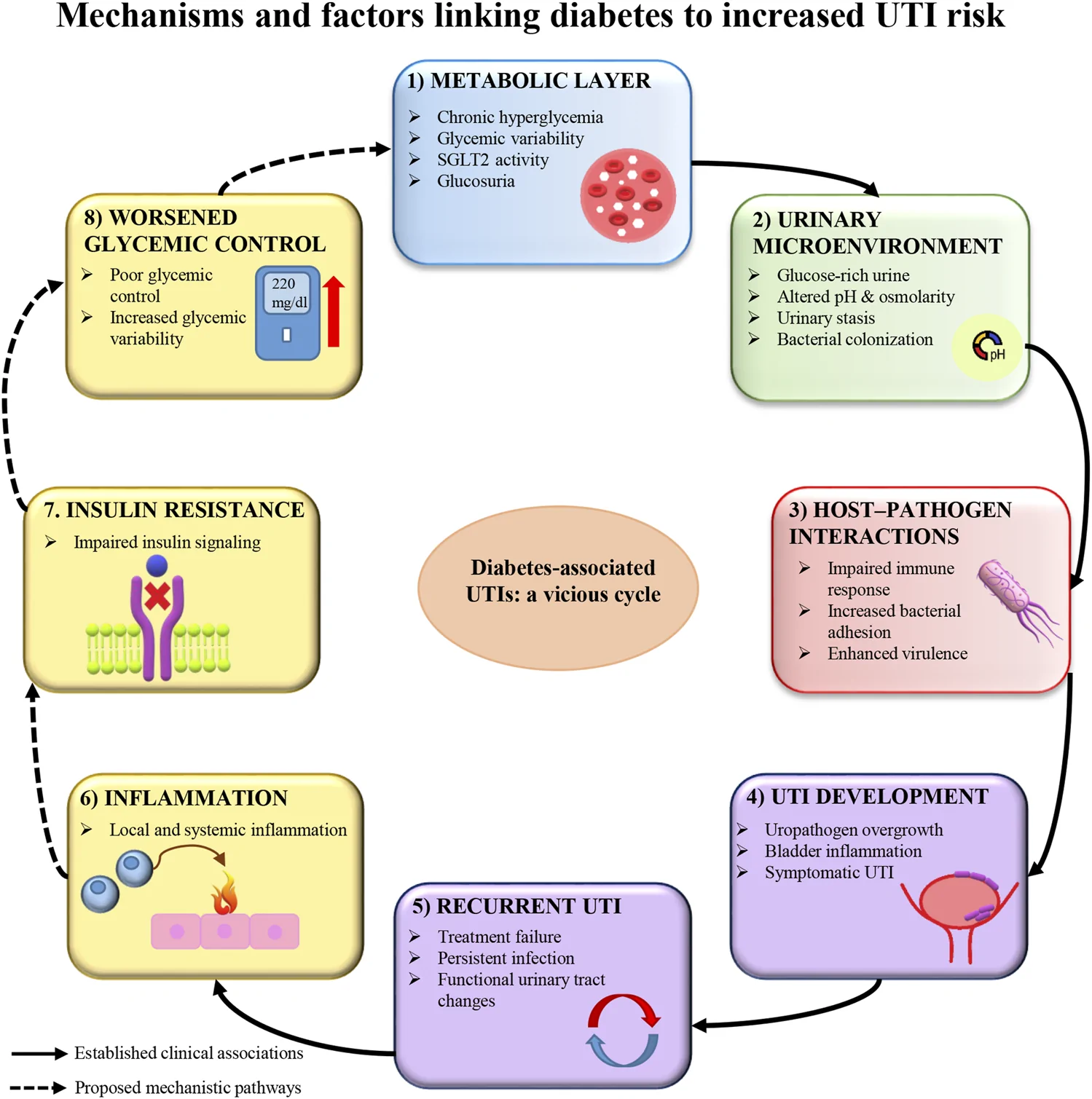
Integrated model depicting the complex interplay among metabolic, urinary, inflammatory, and host–pathogen factors that contribute to the increased susceptibility of individuals with diabetes to UTIs. Persistent hyperglycemia and glycemic variability promote glucosuria and metabolic alterations, resulting in changes to the urinary tract microenvironment that favor bacterial colonization while compromising host immune responses. Consequently, these processes increase the likelihood of symptomatic and recurrent UTIs, as well as sustained inflammation. In turn, chronic inflammation may exacerbate insulin resistance and impair glycemic control, creating a self-reinforcing vicious cycle. Solid arrows denote relationships supported by clinical evidence, whereas dashed arrows indicate proposed mechanistic pathways. Based on Hu et al. (2022), Islam et al. (2022), Mohanty et al. (2022), Confederat et al. (2023), Pishdad et al. (2024), and Ahmed Khalaf (2025).

Hyperglycemia and glycosuria may strongly influence bacterial physiology by enhancing bacterial metabolism, adhesion, and biofilm formation (Islam et al., 2022; Eskandar, 2026). Biofilms are associated with increased tolerance to antibiotics and host immune defenses (Naziri et al., 2021). At the same time, high glucose levels may disrupt epithelial barrier integrity and impair innate immune responses, including neutrophil function and the expression of antimicrobial peptides such as psoriasin, thereby promoting bacterial persistence (Mohanty et al., 2022; Sonkoue Lambou et al., 2022). An additional challenge is the high prevalence of multidrug-resistant UPEC strains in patients with diabetes. Many isolates produce ESBL enzymes including CTX-M, TEM, and SHV-type β-lactamases, encoded by the corresponding resistance genes *bla*_*CTX-M*_, *bla*_*TEM*_, and *bla*_*SHV*_. Resistance to fluoroquinolones and other antibiotic classes is also common (Nayaju et al., 2021; Islam et al., 2022; Sonkoue Lambou et al., 2022). These combined mechanisms contribute to increased severity, recurrence, and therapeutic difficulty of UPEC infections in diabetes (Figure 1) (Chan et al., 2024).

In addition to metabolic alterations, diabetes profoundly reshapes host immune responses, which may result in a state of impaired pathogen clearance and dysregulated inflammation. Defects in neutrophil function, including reduced chemotaxis, phagocytosis, and oxidative burst, compromise the initial innate immune response to UPEC infection (Sonkoue Lambou et al., 2022). At the same time, hyperglycemia-driven formation of advanced glycation end products (AGEs) and subsequent activation of the receptor for advanced glycation end products (RAGE) signaling pathway amplify nuclear factor kappa B (NF-κB)–mediated cytokine production, sustaining a chronic pro-inflammatory milieu that fails to effectively eradicate bacteria while promoting tissue damage (Ahmed Khalaf, 2025). This paradoxical combination of immune suppression and hyperinflammation appears to represent an important determinant of infection persistence in diabetic hosts.

The synergistic effects of bacterial metabolic adaptation, biofilm formation, immune evasion, and host immune dysfunction may create a permissive environment for persistent and recurrent infections in patients with diabetes (Table 1). These interconnected mechanisms are likely to contribute to increased disease severity and therapeutic challenges associated with UPEC infections in diabetes (Islam et al., 2022; Chan et al., 2024). Nevertheless, several mechanisms discussed above are supported primarily by *in vitro* experiments or animal models rather than prospective clinical studies (Tseng et al., 2018; Islam et al., 2022; Mohanty et al., 2022; Neupane et al., 2026). Although these models provide valuable mechanistic insight, their direct translation to human disease should be interpreted with caution. Further clinical studies are required to determine the relative contribution of individual bacterial virulence factors and host immune alterations to infection susceptibility in diabetic patients.

**TABLE 1:** Pathophysiological mechanisms linking diabetes mellitus and UTI.

Mechanism category	Specific alteration in diabetes	Underlying mechanism	Effect on UTI pathogenesis	Clinical implications	References
Metabolic factors	Chronic hyperglycemia	Sustained elevation of blood glucose impairs neutrophil chemotaxis, phagocytosis, and intracellular killing, while disrupting cytokine signaling pathways	Reduced capacity to effectively eliminate invading uropathogens	Increased susceptibility to both initial and recurrent infections	Sorescu et al. (2024), Tang et al. (2025)
	Glycemic variability	Fluctuating glucose levels enhance oxidative stress and promote pro-inflammatory responses, leading to instability of immune function	Inconsistent and impaired host defense mechanisms	Increased risk of infection onset and disease progression	Galindo et al. (2020)
Urinary factors	Glucosuria	Exceeding the renal glucose threshold results in glucose excretion into urine, creating a nutrient-rich environment that supports uropathogen proliferation	Enhanced bacterial growth and survival within the urinary tract	Increased bacterial burden and higher recurrence rates	Confederat et al. (2023)
	Altered urine composition	Changes in urinary osmolarity, pH, and solute composition modify the local microenvironment, facilitating bacterial adaptation and persistence	Improved bacterial survival under urinary conditions	Chronic colonization and reduced treatment efficacy	Pishdad et al. (2024)
Bladder dysfunction	Autonomic neuropathy	Diabetic autonomic neuropathy impairs bladder innervation, leading to decreased detrusor contractility and incomplete bladder emptying	Urinary retention and stasis promote bacterial accumulation	Increased risk of recurrent and complicated UTIs	Ahmad et al. (2020)
Epithelial factors	Uroepithelial dysfunction	Hyperglycemia disrupts epithelial barrier integrity, alters receptor expression, and weakens intercellular junctions, facilitating bacterial adhesion and invasion	Enhanced bacterial attachment and deeper tissue invasion	Greater infection severity and risk of upper tract involvement	Mohanty et al. (2022)
Immune dysfunction	Reduced antimicrobial peptides (e.g., psoriasin)	Hyperglycemia downregulates key innate immune mediators responsible for controlling bacterial growth at mucosal surfaces	Increased bacterial survival at the epithelial interface	Persistent and recurrent infections	Mohanty et al. (2022)
	Chronic inflammation	Sustained activation of pro-inflammatory cytokines (e.g., IL-6, TNF-α) leads to immune dysregulation and tissue damage without effective pathogen clearance	Prolonged but ineffective inflammatory response	Increased risk of complications, including pyelonephritis	Ahmed Khalaf (2025)
Microbial interactions	Enhanced UPEC virulence	Elevated glucose levels act as metabolic substrates and signaling cues that upregulate virulence-associated genes involved in adhesion, invasion, and biofilm formation	Increased bacterial persistence and resistance to host defenses	Higher recurrence rates and increased likelihood of treatment failure	Islam et al. (2022), Tama et al. (2025)
Therapy-related factors	SGLT2 inhibitor use	Pharmacological inhibition of renal glucose reabsorption increases urinary glucose excretion, thereby altering the urinary microenvironment	Enhanced availability of glucose for bacterial growth	Potentially increased risk of UTI in selected patient populations	Hu et al. (2022)

## Future perspectives and therapeutic implications

Advances in understanding the molecular and cellular mechanisms underlying UPEC infections in patients with diabetes have identified several potential therapeutic targets. Because hyperglycemia not only impairs host defense mechanisms but also enhances bacterial virulence, effective glycemic control remains an essential component of infection management. Improved metabolic regulation may reduce glycosuria-associated growth, restore antimicrobial peptide expression, and partially normalize immune responses (Mohanty et al., 2022; Sonkoue Lambou et al., 2022).

Targeting biofilm formation represents another important therapeutic approach. Biofilms contribute significantly to the persistence and recurrence of infection, particularly under hyperglycemic conditions, where UPEC exhibits enhanced biofilm production associated with curli fibers, pili, and increased resistance to antibiotics and host immune defenses (Shahin et al., 2019; Islam et al., 2022). Consequently, disruption of biofilm architecture or inhibition of pathways involved in biofilm formation may improve treatment efficacy.

Modulation of host immune responses has also emerged as a promising strategy. Hyperglycemia-induced downregulation of antimicrobial peptides, including psoriasin, impairs epithelial defense mechanisms and promotes bacterial persistence (Mohanty et al., 2022). Moreover, targeting inflammatory pathways such as the AGE–RAGE–NF-κB axis may reduce tissue damage while preserving antimicrobial activity (Ahmed Khalaf, 2025). Anti-virulence therapies aimed at inhibiting bacterial toxins, adhesion factors, and immune evasion mechanisms may further reduce pathogenicity without increasing selective pressure for antibiotic resistance (Shahin et al., 2019).

The increasing prevalence of multidrug-resistant UPEC strains, including ESBL-producing isolates carrying *bla*_*CTX-M*_, *bla*_*TEM*_, and *bla*_*SHV*_ resistance genes, respectively, emphasizes the need for alternative therapeutic approaches (Nayaju et al., 2021; Islam et al., 2022). In this context, bacteriophage therapy, microbiome-based interventions, and probiotic strategies represent promising adjunctive options (Łukasiak et al., 2026). Future studies integrating genomics, transcriptomics, and metabolomics may provide deeper insight into host–pathogen interactions in diabetes and facilitate the identification of novel therapeutic targets associated with hyperglycemia-driven immune dysfunction and bacterial adaptation (Li et al., 2024).

## Discussion

UTIs in patients with diabetes mellitus represent a significant clinical burden due to their increased incidence, recurrence, and risk of complications (Geerlings, 2008; Nitzan et al., 2015; Kamei and Yamamoto, 2021). The bidirectional relationship between infection and glycemic control complicates disease management, as UTIs may worsen metabolic status while poor glycemic control appears to predispose patients to infection (Dandona, 2004; Galindo et al., 2020; Wajid et al., 2025). This interplay highlights the importance of early recognition of infection in cases of unexplained deterioration in glucose control and the need for coordinated metabolic and antimicrobial management.

An additional challenge is the growing prevalence of antimicrobial resistance among uropathogens in diabetic populations, which limits therapeutic options and underscores the importance of culture-guided therapy (Nayaju et al., 2021; Islam et al., 2022; Sonkoue Lambou et al., 2022). Recurrent antibiotic exposure further contributes to the selection of resistant strains, emphasizing the need for rational antimicrobial use (Abdelaziz et al., 2026).

Although considerable progress has been made in elucidating the complex relationship between diabetes and UTIs, several important knowledge gaps remain. Much of the current mechanistic understanding is based on experimental studies, whereas prospective human studies investigating host–pathogen interactions are still limited (Geerlings and Hoepelman, 1999; Islam et al., 2022; Mohanty et al., 2022). Furthermore, heterogeneity in study design, patient characteristics, diabetes type, glycemic control, and diagnostic criteria complicates direct comparisons across studies and may contribute to inconsistent findings regarding the relative roles of hyperglycemia and glucosuria in UTI susceptibility (Geerlings, 2008; Nitzan et al., 2015; Papp and Zimmern, 2023). Addressing these limitations will be crucial for improving the interpretation of current evidence and establishing more precise models of UTI susceptibility in patients with diabetes.

Future strategies should focus on improving individualized patient management, integrating optimized glycemic control with targeted antimicrobial therapy and risk assessment (Pasquel et al., 2021; Korytkowski et al., 2022). Emerging approaches, including anti-virulence strategies and biofilm-targeted interventions, may offer additional therapeutic potential (Tseng et al., 2018; Islam et al., 2022). A better understanding of host–pathogen interactions in the context of metabolic dysregulation remains essential for improving prevention and treatment outcomes in this population (Li et al., 2024; Neupane et al., 2026).
